# A comparison of methods to adjust for continuous covariates in the analysis of randomised trials

**DOI:** 10.1186/s12874-016-0141-3

**Published:** 2016-04-11

**Authors:** Brennan C. Kahan, Helen Rushton, Tim P. Morris, Rhian M. Daniel

**Affiliations:** Pragmatic Clinical Trials Unit, Queen Mary University of London, London, UK; University of Manchester, Manchester, UK; MRC Clinical Trials Unit at UCL, London School of Hygiene and Tropical Medicine, London, UK; Medical Statistics Department, London School of Hygiene and Tropical Medicine, London, UK

**Keywords:** Randomised controlled trial, Covariate adjustment, Continuous variables, Fractional polynomials, Restricted cubic splines

## Abstract

**Background:**

Although covariate adjustment in the analysis of randomised trials can be beneficial, adjustment for continuous covariates is complicated by the fact that the association between covariate and outcome must be specified. Misspecification of this association can lead to reduced power, and potentially incorrect conclusions regarding treatment efficacy.

**Methods:**

We compared several methods of adjustment to determine which is best when the association between covariate and outcome is unknown. We assessed (a) dichotomisation or categorisation; (b) assuming a linear association with outcome; (c) using fractional polynomials with one (FP1) or two (FP2) polynomial terms; and (d) using restricted cubic splines with 3 or 5 knots. We evaluated each method using simulation and through a re-analysis of trial datasets.

**Results:**

Methods which kept covariates as continuous typically had higher power than methods which used categorisation. Dichotomisation, categorisation, and assuming a linear association all led to large reductions in power when the true association was non-linear. FP2 models and restricted cubic splines with 3 or 5 knots performed best overall.

**Conclusions:**

For the analysis of randomised trials we recommend (1) adjusting for continuous covariates even if their association with outcome is unknown; (2) keeping covariates as continuous; and (3) using fractional polynomials with two polynomial terms or restricted cubic splines with 3 to 5 knots when a linear association is in doubt.

## Background

Adjustment for prognostic covariates in the analysis of randomised controlled trials (RCTs) can offer substantial benefits [1–12]. These include increased power [1–6], protection against chance imbalances between treatment arms [1], and correct results when the covariate was used as a stratification factor during randomisation [1, 8–12]. Adjustment for binary or categorical covariates is relatively straightforward through the use of indicator (or dummy) variables, as there is no risk of mispecifying the nature of the association with the outcome. However, adjustment for continuous covariates is more complex, as the shape of this association does need to be specified. For example, we could assume this association is linear, quadratic, logarithmic, or takes on some other form.

When the shape of the association between covariate and outcome is known (due to previous data, or biological or medical reasons), adjustment for continuous covariates is straightforward. However, when the association is unknown the best method of adjustment is unclear. Potential options include dichotomisation or categorisation (grouping the covariate into two or more categories); assuming a linear association between covariate and outcome; or using the data to estimate a potentially non-linear association, for example by using fractional polynomials [13] or restricted cubic splines (RCS) [14, 15].

The issue of adjusting for continuous covariates has been studied in the context of observational, non-randomised studies [13, 16–19], however there has been comparatively little research into this issue in RCTs. The Committee for Proprietary Medicinal Products’ (CPMP) guidance document *Points to Consider on Adjustment for Baseline Covariates* states that *“… in the absence of any well-established prior knowledge about the relationship between the covariates and the outcome (which is often the case in most clinical trials) the model should use a simple form. For example, when the covariate is continuous, then the model might be based on a linear relationship between the covariate and outcome, or on a categorisation of the covariate into a few levels, the number of levels depending upon the sample size”* [20]. However, these simple approaches may lead to misspecification of the association between the covariate and outcome, which can lead to a decrease in power [21]. Allowing for more complex associations between covariate and outcome may therefore be useful in order to maximise statistical power.

The goals of this paper are to investigate which methods of adjusting for a continuous covariate in the analysis of a RCT maximise power whilst still retaining correct type I error rates and unbiased estimate of treatment effect, when the true association between covariate and outcome is unknown.

## Methods

### Problems with misspecification

We begin by exploring some of the potential issues that may occur if the association between a continuous covariate and the outcome is misspecified (that is, when the assumed association is different to the true association). In general, misspecification will affect results only when there is a true association between covariate and outcome, and for the purposes of this discussion we assume that the covariate *does* influence outcome. It should be noted however that the association between covariate and outcome does not need to be causal. Finally, we only consider covariates measured before randomisation, as adjustment for post-randomisation factors can lead to biased estimates of treatment effect [22, 23].

In observational studies, one of the primary issues with misspecification is residual confounding; that is, adjusting for the misspecified covariate will not fully account for the confounding. This can lead to biased estimates and misleading conclusions. However, residual confounding is not an issue in RCTs, provided the randomisation procedure has been performed correctly, as this ensures there are no systematic differences between treatment arms (although chance imbalances can still occur) [24]. The primary concern regarding misspecification in RCTs is therefore whether it affects the general operating characteristics of the trial, e.g. the estimate of the treatment effect, type I error rate, or power.

Current evidence suggests that misspecification of the covariate-outcome relationship will not increase the type I error rate [21, 25]. However, it can affect power and the estimated treatment effect, though these issues differ for linear vs. non-linear models (where linear models include analyses that estimate a difference in means for continuous outcomes, or a risk difference or risk ratio for binary outcomes, and non-linear models include those that estimate an odds ratio for binary outcomes, or a hazard ratio for time-to-event models with censoring).

For linear models (e.g. a difference in means or proportions), misspecification will not affect the unbiasedness of the estimator of the treatment effect; this remains unbiased regardless of the extent of the misspecification. However, the precision with which the treatment effect is estimated will be reduced, leading to a reduction in power [25].

For non-linear models (e.g. models that estimate odds ratios, or hazard ratios with censoring), misspecification will affect the estimated treatment effect; it will be attenuated towards the null, and power will be reduced [21, 26–28].

In general, the impact will depend on the extent of the misspecification (how far our assumed association is from the true association); greater degrees of misspecification will lead to greater decreases in power, and a higher degree of attenuation in treatment effect for binary or time-to-event outcomes.

### Methods of analysis

**Fig. 1:**
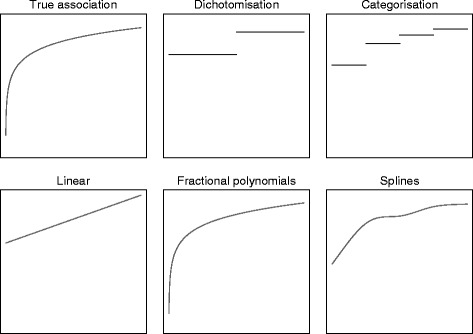
Estimated associations for different methods of analysis for y = log(x). *To obtain the estimated association between f(x) and y for each method of analysis, we first generated a single data set from the model y = log(x) + ε (where ε ~ N(0, 1). We then fit a linear regression model of the form y = f(x) to obtain the estimated association

**Fig. 2:**
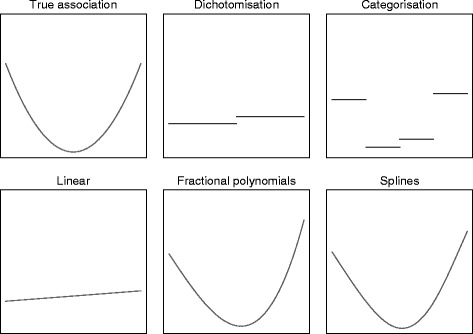
Estimated associations for different methods of analysis for y = x^2^. *To obtain the estimated association between f(x) and y for each method of analysis, we first generated a single data set from the model y = x^2^ + ε (where ε ~ N(0, 1). We then fit a linear regression model of the form y = f(x) to obtain the estimated association

Below we outline various methods to adjust for continuous covariates, and highlight the assumptions made by each analysis. Figures 1 and 2 compare the estimated association for each method of analysis to the true association.

#### Dichotomisation

Dichotomisation involves splitting patients into two groups based on their covariate values. For example, patients may be grouped according to their body mass index (BMI) score as either overweight (25 or over) or not overweight (under 25). As noted by others, this type of grouping can be helpful in clinical practice, but is not necessarily helpful for data analysis [29].

A dichotomised analysis can be implemented using the following model:

$$ Y=\alpha +{\beta}_T{X}_T+{\beta}_O{X}_O+\varepsilon $$

where *X*_*T*_ is a binary variable indicating whether the patient received treatment or control, *β*_*T*_ is the effect of receiving treatment, *X*_*O*_ is a binary covariate indicating whether the patient was overweight (BMI > 25), *β*_*O*_ is the effect of being overweight, and $\varepsilon$ is a random error term.

The primary issue with dichotomisation is that it throws away a large amount of information, which can reduce power. For example, an analysis that treated BMI as continuous would recognise that BMI values of 24 and 26 are different, but much more similar to each other than BMI values of 16 or 34 are. However, a dichotomised analysis (grouped as less or more than 25) treats values of 16 and 24 as identical, values of 26 and 34 as identical, but treats BMI values of 24 and 26 as opposite.

#### Categorisation

Categorisation is when patients are grouped into multiple categories. It is a generalisation of dichotomisation; in this paper we assume categorisation involves three or more groups, in order to distinguish it from dichotomisation.

Like dichotomisation, categorisation reduces the amount of information in the analysis, potentially leading to a loss in power. However, due to the increased number of categories, less information will be lost than in dichotomisation, and it should therefore give better results. For example, categorising BMI into underweight (<18.5), normal weight (18.5 to 24.9), overweight (25 to 29.9), or obese (30 or more) allows BMI scores of 16, 24, 26, and 30 to each have a different effect on outcome, unlike dichotomisation.

A categorised analysis can be implemented using the following model:

$$ Y=\alpha +{\beta}_T{X}_T+{\beta}_N{X}_N+{\beta}_{OV}{X}_{OV}+{\beta}_{OB}{X}_{OB}+\varepsilon $$

where *X*_*N*_, *X*_*OV*_, and *X*_*OB*_ indicate whether the patient is normal weight, overweight, or obese respectively, and *β*_*N*_, *β*_*OV*_, and *β*_*OB*_ are the effects of being in these BMI categories compared to being underweight.

In general, a higher number of categories leads to less information lost [21]. However, having too many categories can be problematic, particularly with a small sample size. This can lead to reduced power due to the extra parameters in the model [1]. It can also lead to inflated type I error rates and biased estimates of treatment effect for binary or time-to-event outcomes when the number of categories is high compared to the number of events [1].

#### Linear association

A linear analysis keeps the covariate as continuous, and assumes the association between covariate and outcome is linear. This analysis assumes the effect of an increase in the covariate is constant across the range of the covariate. For example, an increase in BMI from 15 to 16 would have the same impact on outcome as an increase from 29 to 30.

A linear analysis can be implemented using the following model:

$$ Y=\alpha +{\beta}_T{X}_T+{\beta}_{BMI}{X}_{BMI}+\varepsilon $$

where *X*_*BMI*_ represents BMI on a continuous scale, and *β*_*BMI*_ represents the effect of a one-unit increase in BMI on outcome.

The primary advantage of a linear analysis over categorisation is that it makes full use of the data, and so should increase study power. However, if the true association between covariate and outcome is non-linear, then a linear analysis will be misspecified and may lead to reductions in power.

#### Fractional polynomials

Fractional polynomial models use polynomial transformations to estimate the association between the covariate and outcome. They typically use either one or two polynomial terms. A model using only one polynomial term is referred to as an ‘FP1’ model, and a model using two polynomials terms an ‘FP2’ model. An FP1 can be written as follows:

$$ Y=\alpha +{\beta}_T{X}_T+{\beta}_1{X}_{BMI}^{P1}+\varepsilon $$

where *p*_1_ is a polynomial transformation estimated from the set {−2, −1, −0.5, 0, 0.5, 1, 2, 3} (where *p* = 0 is taken to mean, by convention, log(*X*)).

A model with two polynomial terms (FP2) can be written as:

$$ Y=\alpha +{\beta}_T{X}_T+{\beta}_1{X}_{BMI}^{P1}+{\beta}_2{X}_{BMI}^{P2}+\varepsilon $$

where *p*_1_ and *p*_2_ are polynomial transformations estimated from the set {−2, −1, −0.5, 0, 0.5, 1, 2, 3} (where *p* = 0 corresponds to log(*X*)). By convention, *p*_1_ = *p*_2_ is taken to mean a model in which the two terms are *β*_1_*X*_*BMI*_^*P*1^ and *β*_2_*X*_*BMI*_^*P*2^ log(*X*_*BMI*_).

Either FP1 or FP2 models could be used in practice. It is possible to use the trial data to select between FP1 and FP2 models, however we do not recommend this approach, as covariate selection procedures have been shown to lead to poor results [30]. We therefore recommend pre-specifying the use of either an FP1 or FP2 model, and consider both approaches in this paper. Furthermore, in some software packages fractional polynomials also incorporate a model selection algorithm, where they drop covariates which are not prognostic enough (e.g. in the example above, they may drop BMI from the final model if it does not meet some pre-defined statistical significance threshold). As mentioned earlier, we do not recommend model selection in RCTs, and so for the purposes of this paper we have only considered fractional polynomial models which include all covariates, regardless of their statistical significance.

The benefits of using a fractional polynomial approach include keeping the data as continuous, and allowing for non-linear associations. Fractional polynomials can be implemented in most standard statistical packages (e.g. using the *fp* or *mfp* commands in Stata, or the *mfp* package in R). Further details on fractional polynomials are available elsewhere [13, 31].

#### Restricted Cubic Splines

RCS is implemented by splitting the continuous covariate into separate sections, separated by *m* different knots, *k*_*1*_ *< k*_*2*_ *< … < k*_*m*_. Within each of these sections, a polynomial relationship between the covariate and outcome is estimated; these polynomial functions are joined up at the knots, to ensure a smooth curve across the range of the covariate. Two boundary knots *k*_*min*_ *< k*_*1*_ and *k*_*max*_ *> k*_*m*_ (usually placed at the extremes of the covariate) are also used; RCS estimates a linear association between covariate and outcome in these boundary knots, i.e. between *k*_*min*_ and *k*_*1*_, *and k*_*m*_ and *k*_*max*_.

The model can be written as:

$$ Y={\beta}_T{X}_T+S(BMI)+\varepsilon $$

where

$$ S(BMI)={\gamma}_0+{\gamma}_1{X}_{BMI}+{\displaystyle \sum_{j=1}^m}{\gamma}_j\left[{\left({X}_{BMI}-{k}_j\right)}_{+}^3-{\lambda}_j{\left({X}_{BMI}-{k}_{min}\right)}_{+}^3-\left(1-{\lambda}_j\right){\left({X}_{BMI}-{k}_{max}\right)}_{+}^3\ \right] $$

and

$$ {\left({X}_{BMI}-k\right)}_{+}^3=\left\{\begin{array}{cc}\hfill {X}_{BMI}-k\Big){}^3\hfill & \hfill \Big(\  if\ {X}_{BMI}\ge k\hfill \\ {}\hfill 0\ \hfill & \hfill if\ {X}_{BMI}<k\hfill \end{array}\right. $$

and

$$ {\lambda}_j=\frac{k_{max}-{k}_j}{k_{max}-{k}_{min}} $$

Although seemingly complicated, RCS can easily be implemented in most software packages (e.g. the *mkspline* command in Stata, the *effect* option in SAS, or the *hmisc* package in R). In practice one must specify the number of knots to use, and where to place them. One could estimate the optimal number and location of the knots based on the trial data, but as above, these types of model selection procedures do not always work well for RCTs, and we therefore recommend that these choices are pre-specified. In this paper, we consider both 3 and 5 knots, and placing them at specified percentiles of the data [15].

RCSs have similar benefits to fractional polynomials: they keep the data as continuous, and allow for non-linear associations. Further details on RCSs are available elsewhere [15].

### Simulation study

We performed a simulation study to compare different methods of accounting for a continuous covariate in the analysis of a RCT with both continuous and binary outcomes.

We generated outcomes from the following model:

$$ {Y}_i=\alpha +{\beta}_T{X}_{T_i}+{\beta}_{cov}f\left({X}_i\right)+{\varepsilon}_i $$

where ${X}_{T_i}$ is a binary variable indicating whether the patient received treatment or control, *β*_*T*_ is the effect of receiving treatment, *X*_*i*_ is a continuous covariate, *f* (.) is a transformation, *β*_*cov*_ is the effect of the transformed covariate, and *ε*_*i*_ is a random error term.

For continuous outcomes, we set *ε*_*i*_ to follow a normal distribution with mean 0 and standard deviation *σ*_*e*_, with *σ*_*e*_ equal to 1.

For binary outcomes, we set *ε*_*i*_ to follow a logistic distribution with mean 0 and variance π^2^/3. *Y*_*i*_ then represents a latent continuous outcome, and a binary response was generated as 1 if *Y*_*i*_ > 0 and 0 otherwise. This model implies the *β*’s represent log odds ratios.

For both outcome types we assessed three scenarios for *X*:

- A linear association with the outcome: *f* (*X*) = *X*
- A non-linear, monotonic association with the outcome: *f* (*X*) = *e*^*X*^
- A non-linear, non-monotonic association with the outcome: *f* (*X*) = *X*^2^

For each scenario we generated $X$ from a normal distribution with mean 0 and standard deviation 1.

We chose *β*_*cov*_ based on the following formula:

$$ {\beta}_{cov}\left({p}_{90}-{p}_{10}\right)={\sigma}_e $$

where *p*_*z*_ is the *z*^th^ percentile of *f*(*X*); that is, an increase from the 10^th^ to the 90^th^ percentile in *f*(*X*) would increase the outcome by one unit of *σ*_*e*_. For continuous outcomes, this led to *β*_*cov*_ values of 0.385, 0.300, and 0.372 for linear, non-linear monotonic, and non-linear non-monotonic associations respectively. For binary outcomes, this led to *β*_*cov*_ values of 0.700, 0.550, and 0.674 for linear, non-linear monotonic, and non-linear non-monotonic associations respectively.

We set the sample size to 200 patients for continuous outcomes, and to 600 for binary outcomes. These values were selected based on a review of trials published in high impact general medical journals which found these were the median sample sizes for trials with each outcome type [8]. Patients were randomised to one of two treatment arms using simple randomisation. For each simulation scenario (linear, monotonic, non-monotonic) we used two treatment effects: *β*_*T*_ was set to 0, or *β*_*T*_ was set to give 80 % power based on the sample size (assuming correct specification of the association between covariate and outcome). For binary outcomes we set the event rate in the control arm to 50 %. *β*_*T*_ was set to give 80 % power based on both the sample size and the effect of *β*_*cov*_ on outcome; because the effect of *β*_*cov*_ differed according to the scenario, this implies that *β*_*T*_ was set to different values depending on the type of association between covariate and outcome.

We analysed continuous outcomes using a linear regression model, and binary outcomes using a logistic regression model. We adjusted for the continuous covariate *X* in the regression model using seven different approaches: (a) dichotomising *X* at its sample median; (b) categorising *X* at the 25^th^, 50^th^, and 75^th^ sample percentiles; (c) including *X* as a continuous covariate, assuming a linear association; (d) using fractional polynomials, with one polynomial term (FP1); (e) using fractional polynomials, with two polynomial terms (FP2); (f) using restricted cubic splines with 3 knots (knots were placed based on Harrell’s recommended percentiles [15]); and (g) using restricted cubic splines with 5 knots (knots were placed based on Harrell’s recommended percentiles [15]).

For each scenario we calculated the bias in the estimated treatment effect, the type I error rate (when *β*_*T*_ = 0) and the power (when *β*_*T*_ ≠ 0). For each simulation scenario we used 5000 replications.

### Re-analysis of MIST2 and APC trials

We applied the different methods of accounting for continuous covariates to the MIST2 and advanced prostate cancer (APC) trials. MIST2 compared four treatments for patients with pleural infection [32]; placebo, tPA, DNase, or tPA + DNase. We focus on the treatment comparison between tPA + DNase vs. placebo for simplicity. We used a logistic regression model to re-analyse the outcome of surgery at three months. Of the 192 patients included in the analysis, 31 (16 %) experienced an event. We adjusted for the size of the patient’s pleural effusion at baseline (continuous covariate), as well as two binary covariates: whether the infection was hospital acquired and whether the infection was purulent. All three covariates were minimisation factors. In our re-analysis, we handled the continuous covariate (size of the patient’s pleural effusion) in eight different ways: (a) we excluded it; (b) we dichotomised it at its sample median; (c) we categorised it at its sample 25^th^, 50^th^, and 75^th^ percentiles; (d) we included it as a continuous covariate, assuming a linear association with outcome; (e) we used an FP1 model; (f) we used an FP2 model; (g) we used RCS with 3 knots; and (h) we used RCS with 5 knots. For the fractional polynomial models, we forced the model to include the covariate regardless of its significance level, and for the RCS models, we placed the knots at the percentiles recommended by Harrell.

The APC trial compared diethyl stilboestrol vs. placebo on overall survival in patients with advanced prostate cancer. We used a Cox regression model to re-analyse the outcome of overall mortality. In our re-analysis we used the dataset supplied by Royston and Sauerbrei [13]. Of 475 patients included in the analysis, 338 (71 %) experienced an event. We adjusted for three continuous covariates: patient weight, tumour size, and stage grade. All three are prognostic factors associated with mortality. We used the same methods of analysis as for the MIST2 trial above. We analysed each of the three continuous covariates using the same method (that is, dichotomised all three, used an FP2 model for all three, etc.).

## Results

### Simulation results for continuous outcomes

All methods of analysis provided unbiased estimates of treatment effect, and correct type I error rates (range 4.7 to 5.7 %) across all scenarios.

**Fig. 3:**
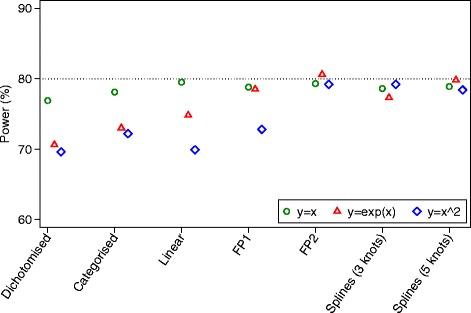
Simulation results for continuous outcomes (power)

Results for power are shown in Fig. 3. For data generated under a linear association, all methods of analysis which kept the covariate *X* as continuous (linear analysis, fractional polynomials, and restricted cubic splines) had nominal power. Conversely, dichotomisation and categorisation led to small reductions in power.

For data generated under a non-linear, monotonic association, FP and splines gave the highest power. A linear analysis led to a reduction in power of about 5.8 % compared to FP2, and dichotomisation and categorisation gave reductions of about 10.0 and 7.6 % respectively.

For data generated under a non-linear, non-monotonic association, FP2 and splines with 3 or 5 knots gave the highest power. A linear analysis and dichotomisation had the lowest power (9.3 and 9.6 % reductions respectively vs. FP2), and categorisation lost 7.0 % power. The FP1 model lost 6.4 % power compared to FP2.

### Simulation results for binary outcomes

**Fig. 4:**
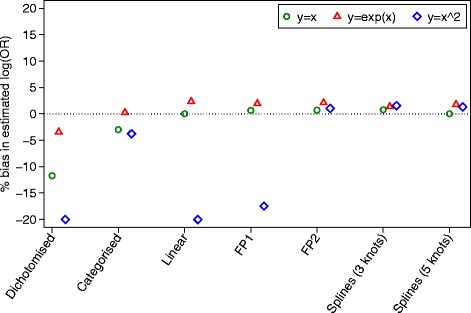
Simulation results for binary outcomes (bias)

**Fig. 5:**
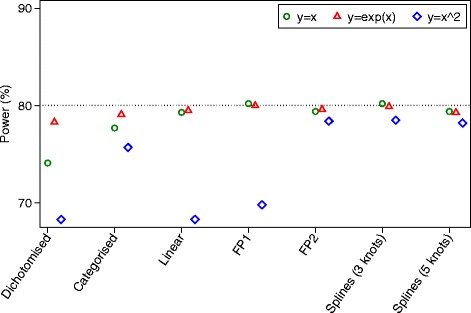
Simulation results for binary outcomes (power)

Results are shown in Figs. 4 and 5. All methods of analysis were unbiased when the treatment had no effect (i.e. when the odds ratio = 1), and gave correct type I error rates (range 4.6 to 5.7 %) across all scenarios. When the treatment was effective (i.e. when the odds ratio ≠ 1), dichotomisation, categorisation, a linear analysis, and FP1 models all led to bias in certain scenarios. This was most pronounced for data generated under a non-linear, non-monotonic association; dichotomisation, a linear analysis, and FP1 all led to the log(OR) being biased downwards by around 20 %. Conversely, FP2 and splines with 3 or 5 knots all produced unbiased estimates across all scenarios. Results are shown in Fig. 4.

For data generated under a linear association, a linear analysis, FP, and splines all gave unbiased estimates of treatment effect and similar power. The log odds ratios for dichotomisation and categorisation were attenuated by 11.7 and 3.0 % respectively, leading to a reduction in power of 5.2 and 1.6 % compared to a linear analysis.

Under a non-linear monotonic association, a linear analysis, FP, and splines all gave unbiased estimates of treatment effect and nominal power. Dichotomisation and categorisation had very little attenuation in the estimated treatment effects, and small reductions in power compared to FP (1.3 % dichotomisation, 0.5 % categorisation).

Under a non-linear, non-monotonic association, FP2 and splines with 3 or 5 knots gave unbiased treatment estimates and good power. Categorisation led to a small attenuation of the estimated log odds ratio, leading to a small decrease in power compared to FP2. A linear analysis and dichotomisation both led to substantially attenuated treatment effects, leading to large decreases in power compared to FP2 (9.1 % linear, 9.3 % dichotomisation). The FP1 model also led to a large degree of bias in the estimated treatment effect, and subsequently a large reduction in power (8.6 %) compared to FP2. This is because FP1 only allows for a monotone association between *X* and *Y*.

### Results of re-analysis of MIST2 and APC trials

Results can be found in Table 1. In both trials, unadjusted/dichotomised/categorised analyses led to smaller treatment effect estimates than linear/FP/spline analyses. Treatment effect estimates were reduced by 35, 24, and 6 % for unadjusted, dichotomised, and categorised analyses respectively, compared to FP or splines in MIST2. This attenuation in the estimated treatment effects led to larger p-values for unadjusted, dichotomised, and categorised analyses in most cases, which sometimes led to results becoming non-significant.

**Table 1:** Results from different methods of adjusting for continuous covariates in APC and MIST2 trials

	APC		MIST2	
Analysis methods	HR (95 % CI)	P-value	OR (95 % CI)	P-value
Unadjusted	0.84 (0.68 to 1.04)	0.11	0.23 (0.05 to 1.15)	0.07
Dichotomised	0.82 (0.66 to 1.01)	0.06	0.21 (0.04 to 1.07)	0.06
Categorised	0.82 (0.66 to 1.01)	0.07	0.18 (0.03 to 0.94)	0.04
Linear	0.79 (0.64 to 0.98)	0.03	0.16 (0.03 to 0.87)	0.03
FP1	0.79 (0.64 to 0.98)	0.03	0.17 (0.03 to 0.90)	0.04
FP2	0.78 (0.63 to 0.97)	0.03	0.17 (0.03 to 0.90)	0.04
Splines (3 knots)	0.80 (0.64 to 0.99)	0.04	0.18 (0.03 to 0.96)	0.04
Splines (5 knots)	0.81 (0.65 to 1.00)	0.06	0.17 (0.03 to 0.91)	0.04

## Discussion

Misspecification of the association between a continuous covariate and the outcome in RCTs can lead to substantial reductions in power. This occurs due to a reduction in the precision of the estimated treatment effect for linear analyses (such as continuous outcomes, or a difference in proportions) and a reduction in the size of the estimated treatment effect for non-linear models (such as a binary or time-to-event outcome with censoring estimated using an odds or hazard ratio). The extent to which results are affected is influenced by the extent of the misspecification.

Re-analysis of the MIST2 and APC trials found that omitting a covariate from the analysis led to larger attenuation of the estimated treatment effect compared to including the covariate, even if the association between covariate and outcome was misspecified. This is likely because excluding a covariate from the analysis can be seen as a more severe form of misspecification, and therefore resulted in larger losses in precision and attenuation of treatment effects. Therefore, we recommend adjusting for covariates even if the true association is unknown.

Our simulation study demonstrated that analyses which keep covariates as continuous generally perform better than analyses that use dichotomisation or categorisation. The simplest method of keeping a covariate continuous is to assume a linear association with the outcome. A linear analysis will perform well if the association between covariate and outcome is approximately linear. However, there may be large reductions in power in the presence of departures from linearity. If non-linearity is possible, then FP2 models or splines are both suitable options, as they have been shown to increase power compared to alternative methods. FP1 models should be used with caution, as these provide very poor results under non-linear, non-monotonic associations. This is because FP requires all covariates to take only positive values, and therefore the outcome is a monotone function of the covariates for any FP1 model.

One issue to consider when deciding if a covariate is likely to have a linear association with outcome is the expected range of the covariate within the trial population. This range will often be smaller in trials than in observational studies due to more restrictive inclusion/exclusion criteria. Covariates with a non-linear association across their entire range may actually have a linear or approximately linear association across certain subsets of their range. For example, imagine BMI is non-linear across the range 16–35. Then, for any small portion of this range (e.g. 16–20, 20–24, etc.), the association may be at least approximately linear. Therefore, in a trial recruiting only overweight patients (BMI 25–29.5), a linear analysis may be appropriate.

Continuous covariates are often categorised when used as stratification factors during randomisation. Stratification or minimisation induces correlation between treatment arms, and it is therefore necessary to account for this correlation in the analysis to obtain valid standard errors and type I error rates [8, 9]. In practice, this means we must include the stratification factors in our analysis. It is therefore of interest to know whether we must use the (categorised) stratification factor, or whether we can use the continuous version. Theoretically, correctly modelling the functional form of the continuous covariate in the analysis should adequately account for the correlation induced by the stratified randomisation procedure, and so this approach should lead to valid standard errors and type I error rates, as well as increased power. However, further research to confirm this hypothesis would be useful.

Both fractional polynomials and restricted cubic splines require certain decisions to be made about their implementation (e.g. FP1 or FP2, number and placement of knots, etc.), and in practice, we could use the trial data to make these choices. For example, one could estimate the optimal number and location of the knots. However, when using trial data to select the model, there is a risk of model overfitting, which can lead to poor results. We therefore suggest that model selection be kept to a minimum, and that the form of the covariates be pre-specified [33, 34]. In addition to pre-specifying the general analysis approach (e.g. assuming a linear association vs. fractional polynomials vs. restricted cubic splines), it is necessary to pre-specify the implementation of these approaches. For fractional polynomials, this entails pre-specifying the whether an FP1 or FP2 model will be used. For restricted cubic splines, this entails pre-specifying the number and location of the knots. We also note that both fractional polynomials and RCS can be combined with model selection algorithms which determine which covariates should be kept in the final model, and which covariates should be discarded (usually based on a statistical significance threshold). However, analysis methods which rely on p-values to determine the form of the final model have been shown to give poor results in RCTs in a variety of scenarios [30, 35–37], and so we do not recommend this approach. Instead, we recommend when using fractional polynomials or splines that all covariates are included in the model regardless of their statistical significance.

## Conclusion

We recommend (1) adjusting for continuous covariates even if their association with outcome is unknown; (2) keeping covariates as continuous; and (3) using fractional polynomials with two polynomial terms or restricted cubic splines with between 3–5 knots when a linear association is in doubt.

### Ethics approval and consent to participate

Not applicable.

### Consent for publication

Not applicable.

### Availability of data and materials

Data from the APC trial are available in *Royston P, Saurbrei W. Multivariable Model-Building. Chichester, England. : Wiley; 2008.* The authors of this manuscript do not have permission to share data from the MIST2 trial.

## Abbreviations

APC: Advanced prostate cancer
BMI: Body mass index
CPMP: Committee for Proprietary Medicinal Products
FP: Fractional polynomial
MIST2: The second multicentre intrapleural sepsis trial
RCT: Randomised controlled trial
RCS: Restricted cubic splines

## References

[1] Kahan BC, Jairath V, Dore CJ, Morris TP (2014). The risks and rewards of covariate adjustment in randomized trials: an assessment of 12 outcomes from 8 studies. Trials.

[2] Hernandez AV, Eijkemans MJ, Steyerberg EW (2006). Randomized controlled trials with time-to-event outcomes: how much does prespecified covariate adjustment increase power?. Ann Epidemiol.

[3] Hernandez AV, Steyerberg EW, Habbema JD (2004). Covariate adjustment in randomized controlled trials with dichotomous outcomes increases statistical power and reduces sample size requirements. J Clin Epidemiol.

[4] Pocock SJ, Assmann SE, Enos LE, Kasten LE (2002). Subgroup analysis, covariate adjustment and baseline comparisons in clinical trial reporting: current practice and problems. Stat Med.

[5] Turner EL, Perel P, Clayton T, Edwards P, Hernandez AV, Roberts I (2012). Covariate adjustment increased power in randomized controlled trials: an example in traumatic brain injury. J Clin Epidemiol.

[6] Thompson DD, Lingsma HF, Whiteley WN, Murray GD, Steyerberg EW (2015). Covariate adjustment had similar benefits in small and large randomized controlled trials. J Clin Epidemiol.

[7] Nicholas K, Yeatts SD, Zhao W, Ciolino J, Borg K, Durkalski V (2015). The impact of covariate adjustment at randomization and analysis for binary outcomes: understanding differences between superiority and noninferiority trials. Stat Med.

[8] Kahan BC, Morris TP (2012). Reporting and analysis of trials using stratified randomisation in leading medical journals: review and reanalysis. BMJ.

[9] Kahan BC, Morris TP (2012). Improper analysis of trials randomised using stratified blocks or minimisation. Stat Med.

[10] Kahan BC, Morris TP (2013). Adjusting for multiple prognostic factors in the analysis of randomised trials. BMC Med Res Methodol.

[11] Kahan BC, Morris TP (2013). Assessing potential sources of clustering in individually randomised trials. BMC Med Res Methodol.

[12] Parzen M, Lipsitz S, Dear K (1998). Does clustering affect the usual test statistics of no treatment effect in a randomized clinical trial?. Biom J.

[13] Royston P, Saurbrei W (2008). Multivariable Model-Building.

[14] Durrleman S, Simon R (1989). Flexible regression models with cubic splines. Stat Med.

[15] Harrell FE (2001). Regression modeling strategies: with applications to linear models, logistic regression, and survival analysis.

[16] Brenner H, Blettner M (1997). Controlling for continuous confounders in epidemiologic research. Epidemiology (Cambridge, Mass).

[17] MacCallum RC, Zhang S, Preacher KJ, Rucker DD (2002). On the practice of dichotomization of quantitative variables. Psychol Methods.

[18] Royston P, Altman DG, Sauerbrei W (2006). Dichotomizing continuous predictors in multiple regression: a bad idea. Stat Med.

[19] Sauerbrei W, Royston P, Binder H (2007). Selection of important variables and determination of functional form for continuous predictors in multivariable model building. Stat Med.

[20] Committee for Proprietary Medicinal Products (CPMP) (2004). Points to consider on adjustment for baseline covariates. Stat Med.

[21] Schmoor C, Schumacher M (1997). Effects of covariate omission and categorization when analysing randomized trials with the Cox model. Stat Med.

[22] Berger VW (2004). Valid adjustment of randomized comparisons for binary covariates. Biom J.

[23] Rosenbaum PR (1984). The Consequences of Adjustment for a Concomitant Variable That Has Been Affected by the Treatment. J Roy Stat Soc a Sta.

[24] Rosenberger WF, Lachin JM (2005). Randomization in Clinical Trials.

[25] Yang L, Tsiatis A (2001). Efficiency Study of Estimators for a Treatment Effect in a Pretest-Posttest Trial. The American Statistician.

[26] Gail M, Wieand S, Piantadosi S (1984). Biased estimates of treatment effect in randomized experiments with nonlinear regressions and omitted covariates. Biometrika.

[27] Hauck WW, Anderson S, Marcus SM (1998). Should we adjust for covariates in nonlinear regression analyses of randomized trials?. Control Clin Trials.

[28] Robinson LD, Jewell NP (1991). Some surprising results about covariate adjustment in logistic regression models. Int Stat Rev.

[29] Altman DG, Royston P (2006). The cost of dichotomising continuous variables. BMJ.

[30] Raab GM, Day S, Sales J (2000). How to select covariates to include in the analysis of a clinical trial. Control Clin Trials.

[31] Morris TP, White IR, Carpenter JR, Stanworth SJ, Royston P (2015). Combining fractional polynomial model building with multiple imputation. Stat Med.

[32] Rahman NM, Maskell NA, West A, Teoh R, Arnold A, Mackinlay C (2011). Intrapleural use of tissue plasminogen activator and DNase in pleural infection. N Engl J Med.

[33] Kahan BC, Jairath V, Murphy MF, Dore CJ (2013). Update on the transfusion in gastrointestinal bleeding (TRIGGER) trial: statistical analysis plan for a cluster-randomised feasibility trial. Trials.

[34] Chan AW, Tetzlaff JM, Gotzsche PC, Altman DG, Mann H, Berlin JA (2013). SPIRIT 2013 explanation and elaboration: guidance for protocols of clinical trials. BMJ.

[35] Freeman PR (1989). The performance of the two-stage analysis of two-treatment, two-period crossover trials. Stat Med.

[36] Kahan BC (2013). Bias in randomised factorial trials. Stat Med.

[37] Shuster JJ (2005). Diagnostics for assumptions in moderate to large simple clinical trials: do they really help?. Stat Med.

